# The Acute Immune Responses of the Common Carp *Cyprinus carpio* to PLGA Microparticles—The Interactions of a Teleost Fish with a Foreign Material

**DOI:** 10.3390/biom12020326

**Published:** 2022-02-18

**Authors:** Ruth Montero, Justin Tze Ho Chan, Bernd Köllner, Roman Kuchta, Jakub Vysloužil, Peter Podhorec, Astrid Sibylle Holzer, Tomáš Korytář

**Affiliations:** 1 Laboratory for Comparative Immunology, Friedrich-Loeffler-Institut, Federal Research Institute for Animal Health, Institute of Immunology, 17493 Greifswald-Insel Riems, Germany; ruth.montero.m@gmail.com (R.M.); bernd.koellner@fli.de (B.K.); 2 Institute of Parasitology, Biology Centre, Czech Academy of Sciences, 370 05 České Budějovice, Czech Republic; justin.chan@paru.cas.cz (J.T.H.C.); krtek@paru.cas.cz (R.K.); astrid.holzer@paru.cas.cz (A.S.H.); 3 Department of Pharmaceutical Technology, Faculty of Pharmacy, Masaryk University, 601 77 Brno, Czech Republic; vyslouzilj@pharm.muni.cz; 4 South Bohemian Research Center of Aquaculture and Biodiversity of Hydrocenoses, Faculty of Fisheries and Protection of Waters, Institute of Aquaculture and Protection of Waters, University of South Bohemia in České Budějovice, 370 05 České Budějovice, Czech Republic; podhorec@frov.jcu.cz

**Keywords:** PLGA, common carp, carrier, teleost fish, antigen, microparticle, aquaculture, vaccine, inflammation, foreign body

## Abstract

Poly lactic-co-glycolic acid (PLGA) particles safely and effectively deliver pharmaceutical ingredients, with many applications approved for clinical use in humans. In fishes, PLGA particles are being considered as carriers of therapeutic drugs and vaccine antigens. However, existing studies focus mainly on vaccine antigens, the endpoint immune responses to these (e.g., improved antibody titres), without deeper understanding of whether fishes react to the carrier. To test whether or not PLGA are recognized by or interact at all with the immune system of a teleost fish, we prepared, characterized and injected PLGA microparticles intraperitoneally into common carp. The influx, phenotype of inflammatory leukocytes, and their capacity to produce reactive oxygen species and phagocytose PLGA microparticles were tested by flow cytometry, qPCR, and microscopy. PLGA microparticles were indeed recognized. However, they induced only transient recruitment of inflammatory leukocytes that was resolved 4 days later whereas only the smallest µm-sized particles were phagocytosed. The overall response resembled that described in mammals against foreign materials. Given the similarities between our findings and those described in mammals, PLGA particles can be adapted to play a dual role as both antigen and drug carriers in fishes, depending on the administered dose and their design.

## 1. Introduction

Whether it is in human patients, laboratory animal models, or reared fishes, carriers offer protection, and timed release of drugs and vaccine antigens in order to produce a desired effect in the host. Several synthetic polymeric particles have been approved by the United States Food and Drug Administration and the European Medicines Agency for applications in humans. Among them, biocompatible and biodegradable poly lactic-co-glycolic acid (PLGA) particles have been tested for delivery of therapies targeting diseases such as type 2 diabetes and prostate cancer [1], whereas research is ongoing in the application of PLGA particles as vaccine antigen carriers [2,3]. Chemically, these particles are polyesters based on lactic and glycolic acids, byproducts of animal metabolism—these components are both the building blocks of PLGA, and the products of PLGA hydrolysis in vivo. Different molar ratios between the components dictate the molecular weight of the copolymer, its hydrophobicity, its degradation, and therefore, the release and delivery of any encapsulated agents [4]. Together, these properties and proven safety and efficacy of PLGA particles make them attractive for therapeutic applications in other (model) organisms.

In fishes, PLGA particles are being considered mainly for delivery of vaccine antigens and other bioactive compounds. Thus far, most studies applied PLGA particles as carriers of antigens in a variety of aquaculture species, including Atlantic salmon [5,6,7,8], rainbow trout [9], Japanese flounder [10], rohu [11], and common carp [12]. While these studies provided encouraging results and demonstrated efficacy, they focus on and measure only the endpoints of immune responses (e.g., antibody titers, survival after challenge) without deeper understanding of the underlying mechanisms [11,12,13]. Furthermore, although these studies point to enhanced capacity of PLGA particles to induce protective immune responses, certain applications, such as hormone [14] or drug delivery, require the opposite of immune activation. Thus, to fully realize the potential of PLGA particles, we must first and foremost study the interaction of PLGA particles with the immune system, since this has broad implications for future applications. 

As several studies have already provided evidence of long-term adjuvant activity and/or tolerance to the particles, here we focus on the short-term acute innate immune response at early timepoints (within days after injection) that dictate outcomes such as the adaptive immune response and the efficacy of any adjuvant/vaccine [15]. 

To test whether the PLGA particles are recognized by the teleost immune system, and to evaluate the extent of induced responses, we used a previously established model of peritoneal stimulation, allowing a fast and accurate evaluation of inflammatory responses in the peritoneal compartment. Thus, the common carp was intraperitoneally (IP) injected with ‘blank’ PLGA microparticles, followed by the analysis of leukocyte kinetics, effector functions and changes in the expression of key inflammatory cytokines. Although we observed that the PLGA microparticles recruited inflammatory leukocytes, the kinetics and magnitude of the response was notably delayed and weaker than those of fish injected with zymosan A. The latter is a yeast-derived microbe-associated molecular pattern that is insoluble like the PLGA particles but can be recognized by toll-like receptor 2 and induces peritoneal inflammation in cyprinids [16,17]. The particles were also minimally reactive when compared to lipopolysaccharide (LPS)-loaded PLGA microparticles in this study, or compared to rainbow trout reacting to gram-negative bacteria [18]. Additionally, peritoneal leukocytes were able to phagocytose and produce reactive oxygen species (ROS) against the smallest µm-sized particles. These activities were greatly diminished in fish receiving a lower dose of the polymer. Overall, the polymer is indeed recognized by the teleost fish immune system, but the reaction is limited, resolving, and resembles more a foreign body response than innate immune recognition of the PLGA. When we take size and dosage into consideration, PLGA particles can be adapted for dual purposes in teleost fishes: as both vaccine antigen carriers and as drug delivery vehicles.

## 2. Materials and Methods

### 2.1. Ethics Statement

Animal procedures were performed in accordance with Czech legislation (section 29 of Act No. 246/1992 Coll. on Protection of animals against cruelty, as amended by Act No. 77/2004 Coll.). Animal handling complied with the relevant European guidelines on animal welfare (Directive 2010/63/EU on the protection of animals used for scientific purposes) and the recommendations of the Federation of Laboratory Animal Science Associations. The animal experiments have been approved by the Ministry of Education. Approval ID: MSMT-4186/2018-2.

### 2.2. Synthesis of PLGA Microparticles

The main sample used for the study was prepared by a standard solvent evaporation method using simple o/w emulsion. As the PLGA carrier, Resomer^®^ RG 653H (Evonik, Darmstadt, Germany; internal viscosity 0.32–0.44 dL/g) was chosen. Resomer^®^ RG 653H is characterized by a 65:35 lactic:glycolic acid ratio, being a suitable foundation for potential controlled release, and it is also acid-terminated, which is advantageous during particle resuspension. Precisely 800 mg of PLGA polymer was dissolved in 5 mL of dichloromethane (Penta, Prague, Czech Republic). This solution represented the oil phase (o). It was then pre-mixed for 60 s in a homogenizer (Ultra-Turrax T25, Ika Werke, Staufen Im Bresgau, Germany) with 12 g of 1% polyvinyl alcohol aqueous solution (PVA, Mw 31,000–50,000; 98–99% hydrolyzed; Sigma Aldrich, St. Louis, MO, USA) to create a concentrated simple o/w emulsion. The pre-mixing step with homogenizer ensured formation of smaller droplets with PVA acting as an emulsifier. Finally, this fine o/w emulsion was diluted by adding it to a large beaker containing 200 g of 0.1% PVA aqueous solution. A dilution step provided sufficient volume for the oil phase droplets during the subsequent evaporation process and minimized risks of droplets merging. The emulsion was stirred with a mechanical stirrer for two hours to completely evaporate dichloromethane. Formed particles were then collected by centrifugation, resuspended in purified water, and lyophilized.

With a slight adjustment to the procedure above, LPS-loaded particles were prepared to determine if they can serve as antigen/vaccine carriers. The addition consisted of mixing the oil phase with 1.5 g of 40 °C warm 9.1% gelatin solution containing LPS from *E. coli* O55:B5 (Sigma-Aldrich, Saint Louis, MO, USA) (water1 phase) at a concentration of 1 mg/mL, and using the homogenizer to create a primary emulsion w1/o. The rest of the protocol remained the same, using the PVA solutions as water2 phase, microparticles effectively formed from w1/o/w2. 

Before proceeding with in vivo experiments, particle endotoxin levels were measured using the Pierce Chromogenic Endotoxin Quant Kit (Thermo Fisher Scientific, Rockford, IL, USA). We performed the test according to the manufacturer’s instructions with technical triplicates except that all proportions were halved, e.g., 25 µL of samples, 50 µL of Chromogenic Substrate Solution, and 25 µL of Stop Solution were combined. We resuspended particles with endotoxin-free water at the highest concentration (100 mg/mL) intended to be used in vivo. Then, PLGA microparticles were centrifuged at 500× *g* for 5 min before 25 µL of supernatant was tested as an unknown. We determined that contamination levels were below 0.05 EU/mL (endotoxin units per mL) (Figure S1) for ‘blank’ PLGA particles without any encapsulated reagent, whereas endotoxin levels of the PLGA-LPS formulation was comparable to that of the 1 mg/mL solution of LPS that served as a positive control.

### 2.3. PLGA Characterization by Microscopy

The surface morphology and size estimation were examined with scanning electron microscopy (SEM; MIRA3, Tescan Orsay Holding, Brno, Czech Republic) equipped with a secondary electron detector (SED). The samples were mounted on a SEM specimen stub using carbon conductive double-faced adhesive tape (Agar Scientific, Stansted Mountfitchet, UK) and were coated with a 20-nm gold layer using a metal sputter with argon atmosphere (Q150R ES Rotary-Pumped Sputter Coater/Carbon Coater, Quorum Technologies, Lewes, UK). SEM images were obtained at an accelerating voltage of 3 kV.

### 2.4. Size Heterogeneity Analysis 

The heterogeneity of particle sizes was determined by laser diffraction, using Horiba LA-960, measuring appropriate amounts of sample using a refractive index of 1.46. As a distribution base, volume (%) was selected. The measurements were performed in triplicate.

### 2.5. Fish

Specific pathogen-free (SPF) common carp (*Cyprinus carpio*) were reared from peroxide-treated fertilized eggs (700 mg/L for 15 min) in an experimental recirculating system in the animal facility of the Institute of Parasitology, Biology Centre CAS. Fish were kept in separate tanks with UV-irradiated and ozonized water at 21 ± 1 °C; water quality (oxygen, pH, ammonia, nitrite and nitrates) was monitored daily using probes and titration tests. Ammonia levels never surpassed 0.02 mg/L. During the experiment, fish with a mass of approximately 25 g were selected and fed twice a day with a commercial carp diet (Skretting) at a daily rate of 1.5% of their body mass.

### 2.6. In Vivo Stimulation of Fish

To evaluate local inflammation after PLGA injection, 15 fish were IP injected with 100 µL of Gibco RPMI 1640 medium as a negative control (Life Technologies Limited, Paisley, United Kingdom); 14 fish with 200 µg of zymosan A derived from *Saccharomyces cerevisiae* (Sigma-Aldrich, St. Louis, MO, USA); 13 fish with 0.1 mg of poly lactic-co-glycolic acid (PLGA) microparticles; 12 fish with 10 mg of the microparticles. All injections were done in a total volume of 100 µL with RPMI 1640 as a diluent. We used fin cuts to distinguish fish from different groups if they were housed in the same tank. The discrepancy in the number of fish between groups is due to additional fish being injected as a contingency. Additionally, 4 untouched/naïve fish were sampled at day 0 (before any injections) to establish a baseline and to measure the number of resident cells in the peritoneum in the absence of any stimulation.

### 2.7. Fish Sampling and Dissection 

Prior to in vivo stimulation of fish, four fish were dissected for the day 0 timepoint. For the other experimental fish challenged with RPMI, PLGA, or zymosan, they were dissected 1, 2, or 4 days post-stimulation with 4 to 6 fish per timepoint. On each sampling day, a peritoneal lavage with 3 mL of RPMI 1640 medium was performed. We loaded the peritoneal leukocytes onto 25% Percoll (GE Healthcare, Uppsala, Sweden) diluted with RPMI 1640, and centrifuged at 500× *g* for 15 min at 4 °C with gentle acceleration and braking. Afterwards, the supernatant was discarded and the pellet and residual Percoll were washed with 10 mL of fresh RPMI 1640 medium. We then centrifuged cells at 500× *g* for 5 min at 4 °C. The pellet was resuspended in 300 µL of RPMI 1640 medium and the suspension split into three equal parts for downstream assays. 

### 2.8. Flow Cytometry of Peritoneal Leukocytes and Detection of ROS with Dihydrorhodamine 123 (DHR123)

For flow cytometry, we added 100 µL out of 300 µL of the peritoneal cell suspension to 100 µL of NucRed Live 647 ReadyProbes Reagent (Life Technologies Corporation, Eugene, OR, USA), prepared by diluting one ‘drop’ into every mL of RPMI 1640. Cells were incubated for 20 min at 26.5 °C in 5% CO_2_. Then, we measured 10% of the cells by flow cytometry by recording for 20 s at a medium flow rate (60 µL/min) on the BD FACSCanto II (BD Biosciences, Prague, Czech Republic). 

From left to right, we applied the following gating strategy to identify host leukocytes and to differentiate peritoneal leukocyte subpopulations (Figure S2): (1) a plot of side scatter area (SSC-A) vs. forward scatter width (FSC-W) was applied to distinguish erythrocytes (that are contaminants from dissection) from leukocytes; (2) based on the SSC-A and FSC area (FSC-A) properties of PLGA particles in the absence of host cells (Figure S2, second row), we created gates specific for host cells before an (3) APC-A vs. FSC-A plot was used to gate on NucRed Live 647^+^ live nucleated cells that are distinguishable from the polymer; (4) we then excluded doublets through both SSC-A vs. SSC width (SSC-W) and FSC-A vs. FSC height (FSC-H) plots and (5) finally, an SSC-A vs. FSC-A plot was set up to differentiate between lymphocytes (FSC-A^low^ SSC-A^low^), granulocytes (FSC-A^high^ SSC-A^high^) and monocytes/monocytic myeloid cells (FSC-A^high^ SSC-A^low^).

We used the same strategy to identify ROS-producing cells among the leukocyte subsets listed above. Once again, 100 µL out of 300 uL of the peritoneal cell suspension was used for this purpose. To quantify ROS-producing leukocytes, cells were incubated with DHR123 (Sigma-Aldrich, St. Louis, MO, USA) and we measured its oxidation into fluorescent rhodamine 123 (R123), by flow cytometry. DHR123 was prepared and stored according to the manufacturer’s recommendations. The reagent was diluted in RPMI 1640 and added to cell suspensions at a working concentration of 0.25 μg/mL. After a 10-min incubation at 26.5 °C in 5% CO_2_, we added 100 µL of ice-cold PBS prior to flow cytometry measurement on the BD FACSCanto II. As a positive control, pooled peritoneal leukocytes were stimulated in vitro with either 0.5× working dilution Cell Stimulation Cocktail (phorbol 12-myristate 13-acetate [PMA] and ionomycin) (Thermo Fisher Scientific, Carlsbad, CA, USA) or 0.2 mg/mL zymosan A for 10 min, by adding it alongside the DHR123. 

### 2.9. Gene Expression Analysis

To evaluate local inflammation after IP injection with PLGA particles, the expression of cytokine markers *tnfa*, *il1b*, *il6a* and *il10* was measured in peritoneal leukocytes by the reverse transcription quantitative polymerase chain reaction (RT-qPCR). Expression of these target genes were calculated relative to the housekeeping gene *b-actin*. qPCR primer sequences were described previously [19,20,21,22]. We prepared RNA freshly from peritoneal leukocytes using the RNeasy Mini Kit (Qiagen, Hilden, Germany) according to the manufacturer’s instructions. A total of 10 ng of RNA per specimen was subjected to one-step real-time RT-qPCR using the Power SYBR Green RNA-to-CT 1-Step Kit (Thermo Fisher Scientific Baltics UAB, Vilnius, Lithuania) according to the manufacturer’s recommendations, and using the recommended thermocycler program. Technical duplicate measurements were made on the QuantStudio 6 (Applied Biosystems, Foster City, CA, USA). Discrepancies of over 0.5 cycles between technical duplicates were addressed by repeating the same specimens again in a new reaction/plate, adjusting Ct values based on an inter-run calibrator, and retaining only replicates with low standard deviations. The data was analyzed using the 2^−ΔΔCT^ method [23]: within each timepoint, within all experimental groups, individual biological replicate Ct values for each target gene was subtracted by their respective *b-actin* Ct measurements, to obtain the ΔCt value. This value was further subtracted by the mean of the ΔCt values from the RPMI group. 

### 2.10. Microscopy 

We collected total head kidney leukocytes from a healthy fish via 25% Percoll density centrifugation. A total of 500,000 cells were incubated in 100 μL suspensions of 100 mg/mL PLGA microparticles in non-tissue culture-treated V-bottom microtiter plates. Two hours later, we stained the mixture with NucBlue Live ReadyProbes Reagent (Life Technologies Corporation, Eugene, OR, USA) according to the manufacturer’s instructions but included 0.25 μg/mL (working concentration) of DHR123 during the recommended 15-min incubation. Cells were washed once with RPMI and then resuspended in PBS before being added to a slide and covered with a slip.

For electron microscopy, 4 additional animals were injected with the high dose of PLGA. One day later, the peritoneal cavity was washed with RPMI, and cells were pooled from all 4 animals before separation with either 25% Percoll or Ficoll-Paque (Cythra, Sweden AB, Uppsala, Sweden). For the former, the pellet was collected, and the latter, both the pellet and the buffy coat were collected to ensure we would identify denser cells having phagocytosed PLGA particles. Each preparation of separated cells was washed with RPMI before resuspension in 500 μL of RPMI. 200 μL was set aside for flash freezing and transmission electron microscopy (TEM) while the rest were allocated for scanning electron microscopy (SEM).

For SEM the obtained suspension was allowed to settle onto poly-L-lysine-coated glass coverslips, dehydrated, and prepared for classical scanning electron microscope (SEM). A part of the same suspension was also placed on filter paper glued with Tissue-Tek on aluminum stubs. Specimens were then frozen by liquid nitrogen slush and immediately transferred under vacuum into the cryo chamber Cryo ALTO 2500 (Gatan). Then, the sample temperature was raised to −98 °C, and vacuum sublimation was performed for 2 min to decontaminate the surface of the specimens. Finally, the specimens were coated with platinum/palladium at −140 °C for 2 min. The specimens were observed in a field emission scanning electron microscope JEOL 7401F operated at 1 kV.

For TEM, cells were pelleted at 1800× *g* and frozen with a Leica EM PACT2 high pressure freezer (Leica Microsystems, Wetzlar, Germany). Using a Leica AFS (Leica Microsystems), samples were freeze-substituted in 100% acetone containing 2% OsO_4_ for 96 h at −90 °C. The temperature was raised at a rate of 5 °C/h to −20 °C and after 24 h, samples were rinsed in acetone and infiltrated with a graded series of resin (EMbed 812, EMS) solutions (25%, 50%, 75% in acetone) for 1 h each. Cells were infiltrated in pure resin overnight, embedded in fresh resin and polymerized at 60 °C for 48 h. Ultrathin sections were stained with uranyl acetate and lead citrate and examined using a JEOL JEM-1010 microscope. 

### 2.11. Statistics

The data were analyzed using the software Prism 8 (GraphPad Software, San Diego, CA, USA). In the graphs, the data are presented as mean values ± standard deviation (SD). Statistical analyses were conducted on log-transformed relative gene expression data. The statistical test applied for each assay is indicated in their respective figure legends.

## 3. Results

### 3.1. Characterization of Synthesized PLGA Microparticles 

We prepared spherical particles with smooth surfaces, with the exception of small pores around 50 nm in diameter observed by SEM (Figure S3a). Our images suggest a heterogeneous size distribution. This was confirmed by laser diffraction analysis (Figure S3b). The mean size of particles was 21.67 ± 18.50 μm in diameter. Cumulative size distribution values are as follows: 10% of the particles were less than 7.09 μm in length, 50% were less than 17.49 μm and 90% were less than 38.25 μm. However, given that no surfactant was used, the largest measured particles could be clusters formed by the imperfect resuspension of the microparticles. Organoleptically, lyophilized particles appeared as a fine white powder and were easily resuspendable in water or cell culture medium for subsequent experiments.

### 3.2. IP Injected PLGA Microparticles Recruit Host Leukocytes to the Peritoneum 

**Figure 1:**
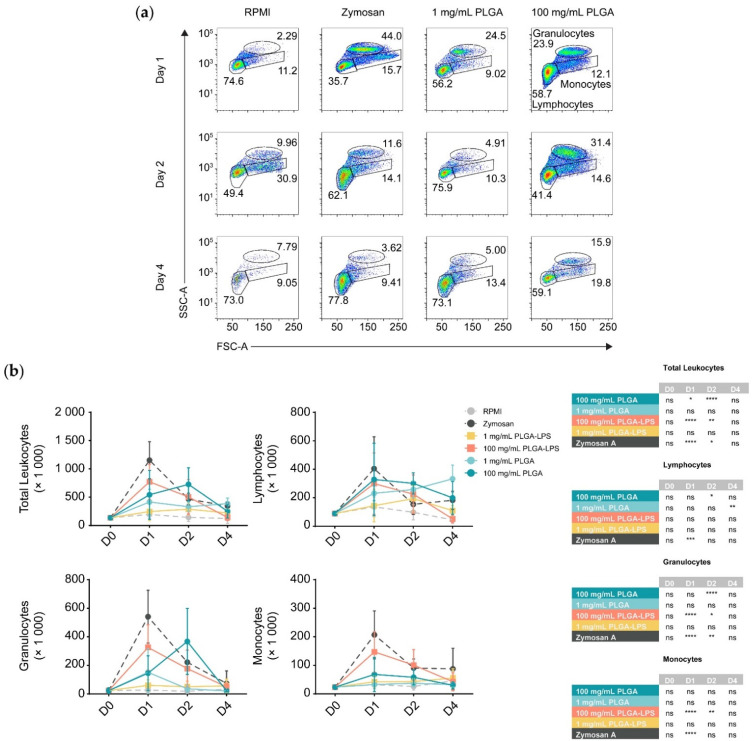
Flow cytometric analysis of peritoneal leukocytes from IP injected common carp with different concentrations of non-encapsulated and LPS-encapsulated PLGA particles. (**a**) Flow cytometry of peritoneal lymphocytes (FSC-A^low^ SSC-A^low^), granulocytes (FSC-A^high^ SSC-A^high^), and monocytes (FSC-A^high^ SSC-A^low^) following different treatments. Each row represents a different timepoint throughout the experiment; the percentages of each leukocyte subpopulation out of total leukocytes are shown. Since acquisition time and rate were identical between specimens, the same proportion of the peritoneal wash was measured (i.e., 33%) from each fish. Thus, the plots presented are comparable and plots with denser cell populations reflect more cell influx into the peritoneum. See Figure S2 for the full gating strategies. (**b**) Graphs summarizing the kinetics of the peritoneal cell influx, after each treatment. The error bars represent the SD (± the mean). We performed a two-way ANOVA along with Dunnett’s multiple comparisons post hoc test, comparing all experimental conditions to the respective RPMI control group at each timepoint; the results are summarized in the table on the right. ns *p* > 0.05; * *p* < 0.05; ** *p* < 0.01; *** *p* < 0.001 and **** *p* < 0.0001.

To determine whether the host reacts to PLGA microparticles introduced by IP injection, we analyzed the composition of peritoneal leukocytes of fish treated with a high (100 mg/mL) or a low dose (1 mg/mL) of PLGA microparticles diluted in RPMI 1640. We monitored for recruitment of leukocytes 1, 2, and 4 days after injection (Figure 1 and Figure S2). These PLGA-exposed fish were compared to fish injected with the particulate pyrogen zymosan A, as well as fish injected with RPMI 1640 cell culture medium as a negative control. Leukocytes were recruited by the microparticles although only the highest concentration of PLGA (100 mg/mL) yielded a significant influx of leukocytes, with the lower concentration (1 mg/mL) being no different from the control group at any timepoint (Figure 1b). Importantly, PLGA did not mimic zymosan A in the kinetics of leukocyte recruitment—the peak of leukocyte recruitment was within a day for the yeast glycan whereas it was a day later for the PLGA. At 4 days post-injection, peritoneal cellularity was comparable between all treatment conditions, indicating that cell efflux occurs and that the acute phase of inflammation is resolved as early as 4 days after injection of PLGA and zymosan A alike. 

During analysis, we broke down leukocytes into individual subpopulations (Figure 1b) to determine the main subsets infiltrating the peritoneum. We again observed differences between PLGA and zymosan A; there was a significant peak of lymphocyte influx at 48 h post-injection using the highest dose of PLGA (100 mg/mL). Using the high dose of PLGA, lymphocytes and granulocytes were the only subsets significantly recruited at any point during the experiment. Interestingly, the lower dose was exceptional, displaying a peak in lymphocyte recruitment 4 days after injection, but was otherwise indistinguishable from RPMI-treated fish. In contrast, inflammation induced by zymosan A was non-selective and recruited lymphocytes, granulocytes, and monocytes equally, peaking 1 day after exposure. Although present, monocytes were never significantly different between PLGA- and RPMI-injected fish.

Furthermore, we prepared and injected LPS-loaded PLGA microparticles to determine whether the differences in the reactivity towards PLGA and zymosan are due to innate immune recognition (e.g., via pattern recognition or toll-like receptors) of zymosan but not the ‘blank’ PLGA. We observed that with the exception of lymphocytes, PLGA-LPS recruited the same leukocyte populations as PLGA without encapsulated LPS, however, the peak of peritoneal cell influx was the same as zymosan A, 1 day post-stimulation (Figure 1b). These effects suggest that the immunostimulatory capacity of PLGA can be further enhanced by addition of toll-like receptor ligands. Overall, based on recruitment of leukocytes into the peritoneum, the PLGA particles alone are indeed recognized by the immune system and not completely inert. However, the acute inflammation they induce is resolved by day 4 after injection. Furthermore, based on the differences in leukocyte recruitment kinetics, the copolymer alone is recognized by the host (immune system) through a different mechanism than PLGA-LPS and zymosan A.

### 3.3. PLGA Microparticles Recruit Inflammatory and ROS-Producing Leukocytes to the Peritoneum

The leukocyte influx we observed in both PLGA- and zymosan-injected fish, suggests that the synthetic polymer may be inducing a host inflammatory response. Therefore, we sought to determine if, in addition to their quantity, the phenotype of recruited leukocytes changed. We first confirmed that the particles induced acute inflammation by measuring changes in expression of both proinflammatory (*tnfa*, *il1b*, and *il6a*) and anti-inflammatory (*il10*) genes in recruited leukocytes. 

**Figure 2:**
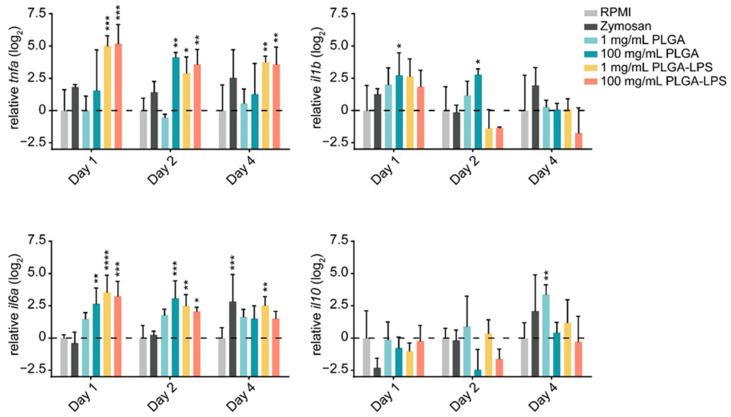
Gene expression profile of peritoneal leukocytes collected from common carp injected with different concentrations of PLGA particles. One graph was prepared for each target gene (either *tnfa*, *il1b*, *il6a*, or *il10*) as indicated by y-axis titles. The units of measure are log-transformed fold-changes in normalized expression of the target gene (i.e., expression of the target gene normalized to the housekeeping gene *b-actin*) of each experimental condition relative to the normalized expression of the same target in the RPMI group, at each timepoint. The data is presented as mean values with SD error bars. A two-way ANOVA was performed with Dunnett’s multiple comparisons *post hoc* test to compare each experimental condition to the respective RPMI group at each timepoint. * *p* < 0.05; ** *p* < 0.01; *** *p* < 0.001 and **** *p* < 0.0001.

The highest dose (100 mg/mL) of PLGA microparticles recruited leukocytes significantly overexpressing and sustaining *il6a* and *il1b* expression during the first 2 days of the experiment compared to the RPMI control group (Figure 2). Although its expression was not different from the negative control group, 1 day post-injection, *tnfa* was significantly overexpressed at the 2-day timepoint. These changes were all resolved by the final day of the experiment. Interestingly, the lower dose of PLGA induced no such changes among the markers of inflammation, but *il10* was overexpressed on the final day of the experiment. Overall, these results confirm that PLGA microparticles have the capacity to induce acute and limited inflammation and this effect is dose-dependent. Interestingly, the expression of *il10* induced by the lowest dose of PLGA particles suggests that it is inversely dose-dependent—*il10* expression trended upward throughout the experiment and was significantly upregulated at the final timepoint (Figure 2). These results suggest that the amount of PLGA microparticles, depending on whether a high or low dose is selected, can induce a qualitative change in the immune response. Overall, the peak of inflammation (at the 2-day timepoint) associated with high doses of PLGA and the distinct profile of the lower dose both correspond to the cell influx we measured by flow cytometry (Figure 1).

The inflammatory reaction to PLGA-LPS microparticles was vastly different. Not only was the peak of expression of inflammatory cytokines *tnfa* and *il6a* observed earlier at the 1-day timepoint, but resolution was not observed by the final timepoint of the experiment in either of the genes. As for the reaction to zymosan, we measured *tnfa* and *il1b* overexpression and *il10* downregulation on day 1. These results suggest that the yeast product induced inflammation at the peak of leukocyte influx, although none of these changes were statistically significant. We therefore confirmed that the PLGA microparticles alone cause only acute inflammation through a distinct mechanism/pathway and that this inflammation is prolonged beyond the initial couple of days if an immunogen is encapsulated into the particles.

**Figure 3:**
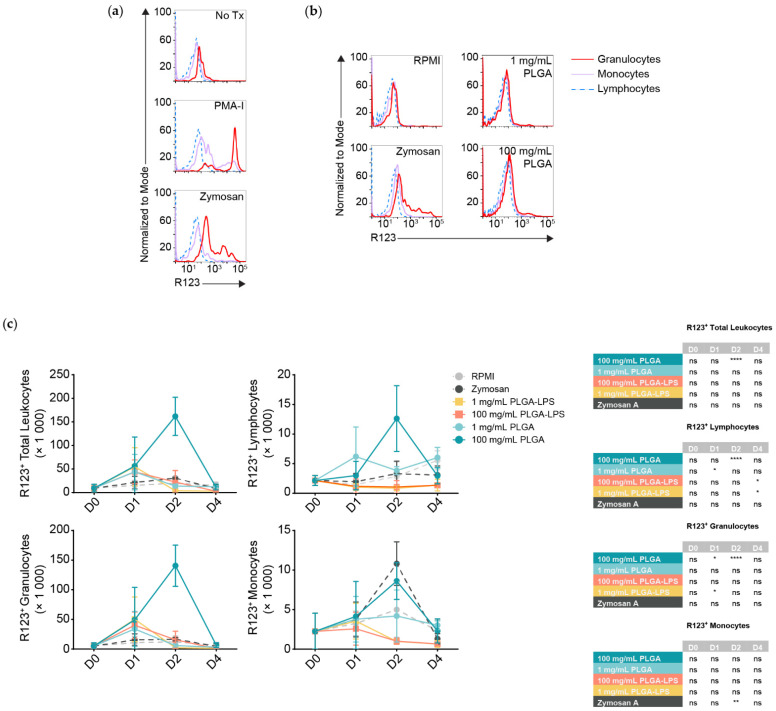
Leukocytes that are recruited to the peritoneum in response to high-dose PLGA particles produce reactive oxygen species (ROS). (**a**) Measurement of ROS production via oxidation of DHR123 to R123 in leukocytes stimulated in vitro with phorbol 12-myristate 13-acetate (PMA-I) or zymosan A. The x-axis represents the intensity of R123, whereas the y-axis represents the frequency of cells normalized as a percentage of the mode at a given R123 intensity (i.e., the number of events/cells at a given R123 intensity divided by the number of events recorded at the modal R123 intensity). Colors represent the different leukocyte populations that we introduced in Figure 1. (**b**) Representative histograms of ROS production in peritoneal leukocytes stimulated in vivo with either RPMI, zymosan, or different doses of blank PLGA. (**c**) Graphs summarizing the number of ROS-producing leukocytes recruited to the peritoneum following intraperitoneal injection with different stimuli at different timepoints. The data is plotted as mean ± SD. Statistical significance was determined using a two-way ANOVA and Dunnett’s multiple comparisons test, relative to the respective RPMI condition at each timepoint. The results of this test are presented in the table organized by different treatment conditions and timepoint. ns *p* > 0.05; * *p* < 0.05; ** *p* < 0.01 and **** *p* < 0.0001.

Although all populations of leukocytes are recruited and together present a proinflammatory phenotype, not all will interact with and react to the microparticles directly. DHR123 has been successfully applied in fishes [24,25], it responds to stimuli such as PMA (Figure 3a) [26], it can identify phagocytes interacting with and responding to particulates such as bacteria, and it is an indicator of antigen breakdown, processing, and presentation [27]. Thus, we decided to measure any respiratory burst activity/ROS production in peritoneal leukocytes by the oxidation of dihydrorhodamine 123 (DHR123) to rhodamine 123 (R123). We stimulated peritoneal leukocytes pooled from healthy fish in vitro using zymosan A and a commercial cell stimulation cocktail, to demonstrate that R123^+^ cells are predominantly myeloid, whereas lymphocytes are either ROS non-producing or do not produce detectable amounts (Figure 3a). Thus, lymphocytes served as an internal control to establish the threshold between R123^+^ and R123^−^ populations among leukocytes of the same individual.

Strikingly, only the highest dose of PLGA (100 mg/mL) led to significant recruitment of R123^+^ leukocytes 2 days after injection (Figure 3c, top left graph) and these results match the peak of cell influx and expression of inflammatory gene markers (Figure 1 and Figure 2). Zymosan A, however, did not recruit more total R123^+^ leukocytes at any timepoint compared to the negative control despite inducing comparable cell influx. We analyzed leukocyte populations separately and observed that, for the 100 mg/mL dose of PLGA, lymphocytes and granulocytes significantly contributed to the peak of total R123^+^ leukocytes seen at the 2-day timepoint (Figure 3c). Granulocytes were not only the major infiltrating subset (Figure 1b), but also the most ROS-producing, numbering at least 10 times more than the lymphocytes and were even significantly different one day after injection of high-dose PLGA. Although not significant, R123^+^ monocytes showed an increase with the 100 mg/mL PLGA 2 days post-injection; with zymosan A, the increase was significantly higher at this timepoint. 

Otherwise, we again observed atypical lymphocyte behavior from the low-dose PLGA group, which presented more R123^+^ lymphocytes one day after exposure. LPS-encapsulated PLGA produced a phenotype more similar to zymosan A than to other conditions, with only sparse recruitment of R123^+^ lymphocytes and granulocytes that do not correspond with the kinetics nor magnitude of leukocyte influx. Overall, our results highlight the contrast between peritoneal leukocytes responding to zymosan A, PLGA-LPS and ‘blank’ PLGA that does not contain any encapsulated reagent. The latter, specifically high-dose ‘blank’ PLGA, was dominant in inducing ROS production from (inflammatory) lymphocytes and granulocytes unlike any other condition, suggesting that there is more beneath the surface of PLGA microparticles than simply the capacity to cause acute inflammation.

### 3.4. Carp Granulocytes Phagocytose PLGA Particles

The contrast between the recruitment of leukocytes that produced ROS in the peritonea of zymosan-, PLGA-LPS- and ‘blank’ PLGA-exposed fish, led us to hypothesize that microparticles were being phagocytosed, and that the ROS we detected was produced in reaction to the particles in phagolysosomes.

**Figure 4:**
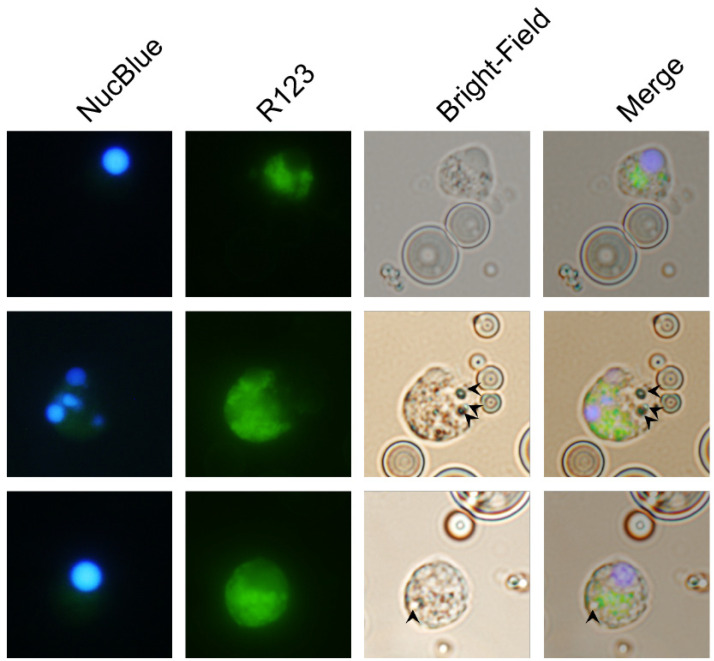
Microphotographs of particle engulfment and concurrent reactive oxygen species production from head kidney leukocytes exposed to PLGA microparticles in vitro. Head kidney leukocytes were isolated from naïve animals and exposed to 100 mg/mL of PLGA microparticles in vitro. Nucleated cells were identified by staining with the NucBlue Live ReadyProbes Reagent, whereas the oxidation of dihydrorhodamine 123 to green-fluorescent rhodamine 123 (R123) was used as an indication of reactive oxygen species production. We either identified cells only producing ROS (**top row**) or concurrently producing ROS along with having internalized PLGA microparticles. The (**middle and bottom rows**) are representatives of ROS-producing polymorphonuclear or monocytic phagocytes, respectively. The black arrows in ‘Bright-Field’ and ‘Merge’ images in the last two rows point towards phagocytosed PLGA microparticles.

We took advantage of the respiratory burst elicited by PLGA microparticles and used fluorescence microscopy to distinguish non-reactive R123^−^ cells from R123^+^ activated cells that have taken up microparticles (visible in bright-field images). As the major hematopoietic lymphoid organ in teleost fishes, the head kidney harbors both undifferentiated and differentiated leukocytes and is the source of circulating and infiltrating leukocytes. Thus, we incubated PLGA microparticles with ex vivo head kidney leukocytes from naïve animals. This approach allowed us to amplify any differences we initially observed without waiting for days for the influx of relevant ROS-producing cell populations in in vivo setups. We identified R123^+^ cells that did not harbor particles, either because inflammation and ROS production are indicators of, but not exclusive to phagocytosis, or perhaps because the particles had been broken down (Figure 4, top row). However, we also identified activated R123^+^ polymorphonuclear (Figure 4, middle row) and monocytic cells (Figure 4, bottom row) that had actively taken up the smallest microparticles, but not the average-sized particle, demonstrating that carp leukocytes are capable of phagocytosing the copolymer and attempting to break it down. Overall, these results together with the flow cytometry of peritoneal leukocytes and the gene expression profiling, suggest that recruited phagocytes (lymphocytes, granulocytes, or monocytes) have the capacity to interact with PLGA microparticles, to contribute to the acute inflammation observed, and potentially to support antigen presentation.

**Figure 5:**
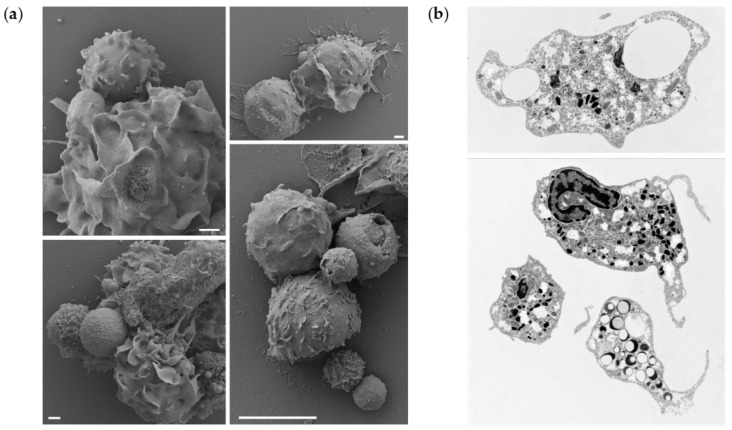
Electron microscopy microphotographs of particles interacting with and being engulfed by peritoneal neutrophils exposed to PLGA microparticles in vivo. Peritoneal leukocytes were collected one day after injection of 100 mg/mL PLGA microparticles. We visualized the cells by either (**a**) scanning electron microscopy (SEM, scale bars represent 1, 1, 1, and 10 μm, respectively) or (**b**) transmission electron microscopy (TEM). (**a**) The SEM shows microparticle-cell interactions, primarily with the neutrophil fraction. Small ingested PLGA particles are visible on the surfaces of the cells. Presence of the particles also induces netosis of inflammatory neutrophils, as evidenced by the PLGA particle captured in the released DNA. (**b**) The microparticles were also detected inside cells, demonstrating that peritoneal phagocytes take up PLGA microparticles in vivo. As shown by the TEM, neutrophils exhibit a high activation stage, with very few granules and a number of empty vesicles surrounding the phagosomes.

To further demonstrate that the microparticle uptake also occurs in vivo, a portion of peritoneal leukocytes from fish exposed to the highest dose of PLGA were studied by electron microscopy, to observe interaction/phagocytosis of the particles (Figure 5a) or reveal sections of phagocytes that have phagocytosed particles (Figure 5b). We observed cells interacting with the microparticles, leading to their uptake and detection of intracellular particles. More specifically, our results suggest that the presence of PLGA particles in the peritoneal cavity initiates a full spectrum of neutrophil activities. We observed not only attachment of the particles to the cell surfaces, their uptake, but also the formation of neutrophil extracellular traps (NETs) surrounding the larger particles. Furthermore, the TEM provided evidence of neutrophil activation, as evidenced by the depletion of intracellular granules, released from the cells upon activation. Collectively, these results indicate that microparticles are taken up in vitro and in vivo.

## 4. Discussion

Based on demonstrated safety and efficacy in other animals, PLGA particles are being translated for therapeutic applications in many fish species. However, there is not enough evidence of how it interacts with the immune system and whether it is a suitable carrier for applications such as drug and vaccine antigen delivery—only the latter benefits from interaction with leukocytes and the immune system. In contrast, from mammalian studies, we know that PLGA particles alone activate human dendritic cells [28,29], but that they can also be inert or at least not cause long-term side effects [13]. How do the particles react in fish? Here, we produced evidence of interactions resembling the foreign body reaction/response observed in mammals, but in a teleost fish. For future translation of PLGA in fishes we need to carefully select and control their parameters during synthesis and application.

### 4.1. Innate Immune Recognition versus a Foreign Body Response

Here, we demonstrated that the fish immune system does indeed recognize and react to PLGA particles but via a different mechanism than the one(s) used to recognize microbes and their products. In contrast to the host reaction against PLGA-LPS and zymosan, the high dose of ‘blank’ PLGA recruited cells whose cellularity peaked 2 days after injection rather than a day after injection. The recruited cells displayed an inflammatory phenotype, overexpressing inflammatory cytokines *tnfa*, *il6a*, and *il1b* but not anti-inflammatory *il10*. Although resolved in PLGA-injected fish, overexpression of *tnfa* and *il6a* were sustained in PLGA-LPS-injected fish. Due to the absence of an endotoxin like LPS or a yeast glycan like zymosan, the particles are unlikely to be readily detectable by the immune system of the host via innate immune pattern recognition or toll-like receptors. 

The cells recruited to the peritoneum also produced ROS. Unlike the gene expression analysis of bulk cell populations, we were able to determine, by applying DHR in a flow cytometric assay, that phagocytes/granulocytes interact with the PLGA particles. In PLGA-LPS-injected fish, the number of ROS-producing peritoneal leukocytes was only a fraction of that from ‘blank’ PLGA-injected fish. The cell influx, ROS production and inflammation, could have peaked in a matter of hours, between the time of injection and the one-day timepoint due to the LPS content. Likewise, it took only minutes for peritoneal leukocytes to react to zymosan or PMA-ionomycin in vitro (Figure 3a). These results are identical to those obtained from human neutrophils exposed to zymosan or PMA and then produced ROS within minutes [27]. We therefore hypothesize that common carp neutrophils were the main ROS producers and subset responding early to a foreign material [30].

Our findings indicate that the response elicited by PLGA microparticles is not identical with that against a microbe or microbial components; it rather resembles the response against a foreign body. In mammals, the foreign body response is one described against synthetic materials such as implanted devices [31]. By design, their size and/or inorganic nature prevents their metabolic breakdown and clearance by the host in the long-term, unlike microorganisms. It involves early adsorption of host proteins and recruitment of neutrophils to the foreign material. Then, macrophages and fibroblasts are recruited sequentially to form fused foreign-body giant cells and fibrotic tissue, respectively, in order to sequester the foreign body. One day after IP injection with the high dose of PLGA, host leukocytes were recruited to the peritoneum. The influx of granulocytes at the peak of the response mimics the rapid influx of neutrophils described in the foreign body response [30]. Along with the recruitment of monocytes/macrophages that we observed, as well as observations of fibrotic tissue in some fish (Figure S4), signs point towards the common carp mounting a foreign body response. 

This study is one of the few to describe a foreign body response in a teleost fish. Compared to a study in which vicryl and nylon sutures were surgically implanted into zebrafish [32], we measured a much stronger recruitment of neutrophils, but we could not observe chronic inflammation nor fused giant cells within the timeframe of our experiment. Another study compared the foreign body response of zebrafish to *Schistosoma mansoni* (a trematode parasite) eggs and to synthetic polyethylene and polystyrene microspheres [33]. Within 6 h post-implantation, macrophages were recruited and adhered to the microspheres (>27 µm and therefore larger than the average particle used in this study). Within 5 days post-implantation, granulomas formed around the microspheres. Such studies and ours support foreign body responses being conserved in fish as in mammals.

### 4.2. Translation of PLGA for Therapeutic Applications in Fishes

For future therapeutic application of PLGA in fishes, production of particles must follow more stringent standards. To meet individual needs, the properties and the application of the particles must be customized: namely size, toxicity/safety, loading capacity, and acid composition as intrinsic properties of PLGA particles; dosage and route of administration.

Here, we produced µm-sized particles that were very diverse in size, ranging from roughly 1 to 40 µm in length. This was beneficial for the purpose of this study because we observed a variety of fish host responses that may each be specific to particles of a certain size: only the smallest particles (1 to 5 µm in size) were phagocytosed, the host did not produce ROS to the larger particles but responded to them as a foreign body, and particle aggregates (due to the lack of a surfactant during particle production) likely promoted fibrosis in the foreign body response (Figure S4). However, for therapeutic application of the particles, such a variety of particles would produce unwanted side effects.

To reproduce only certain effects that we observed in this study (e.g., phagocytosis of particles) but not others, we can manipulate the parameter of particle size. One means to do this is by the choice of organic solvent which impacts the size of the particles and their loading capacity—dissolution in dichloromethane produces larger particles [34], but is less efficient at drug loading than dissolving PLGA in ethyl acetate [35]. Toxicity-wise, we would also benefit from considering alternatives to organic solvents because even residual non-evaporated solvent can alter the immunological profile of host leukocytes. A protocol designed by Nomura et al. (2018) replaces these solvents with vegetable oil for production of Rhodamine B-loaded PLGA microcapsules [36]. Rational design of the particles such as increasing the ratio of lactic acid to glycolic acid can delay the antigen release by weeks [8,37]. Therefore, customizing the properties of microparticles (size, ratio of lactic acid to glycolic acid, amount of antigen encapsulated, etc.) will produce a desired effect in the host. 

Apart from the quality of microparticles, we can also control the quantity introduced and the route of administration. One study demonstrated that antibody titers were dependent on the dose of PLGA-encapsulated antigen [37] whereas another demonstrated the same dependence for cell-mediated cytotoxic responses [38]. If managed strategically, certain applications in drug delivery may suffice with only a minimal amount of particles (determined to be about 0.01 mg per g of fish body mass in this study). Depending on the antigen/drug being delivered, the oral route should also be considered for administration of synthetic polymers [10]. Since PLGA particles can resist the acidity of the stomach, introducing PLGA particles through the oral route can produce distinct effects such as delivering material into the gut-associated lymphoid tissues [39,40]. 

## 5. Future Directions

Now that we have a better understanding of what happens at the local site of injection of PLGA particles, we can further explore which host compartment the particles are delivered to, the systemic effects, antigen-specific responses, and protection against disease, as performed in mammals following administration of PLGA particles [3,41]. At the cellular level, due to the phagocytosis of PLGA particles by granulocytes and phagocytes, we may have the potential to study antigen presentation in these cells [38,42]. To explore these topics, we must proceed with encapsulation of model antigens with demonstrated immunogenicity in fish and to which detection reagents are readily available.

Overall, we have shown that PLGA particles interact with the host immune system. As researchers have done for decades for mammals, the particles can be rationally designed and synthesized to meet individual needs including the needs of the farmers, biologists, parasitologists, immunologists, and others interested in fish health. 

## Data Availability

The data presented in this study are available in the main text, figures, tables and Supplementary Material.

## Supplementary Materials

The following supporting figures can be downloaded at: https://www.mdpi.com/article/10.3390/biom12020326/s1, Figure S1: Endotoxin detection with the Pierce Chromogenic Endotoxin Quant Kit; Figure S2: Gating strategies for distinguishing host cells from PLGA microparticles, and for distinguishing host leukocyte subpopulations; Figure S3: Scanning electron microscopy (SEM) images of the synthesized PLGA microparticles; Figure S4: Fibrotic tissue in the peritoneal cavity of a fish injected 4 days prior with PLGA particles encapsulated with LPS.
