# Supplementary material for: The Acute Immune Responses of the Common Carp Cyprinus carpio to PLGA Microparticles—The Interactions of a Teleost Fish with a Foreign Material
Source: Biomolecules. 2022 Feb 18;12(2):326. doi: 10.3390/biom12020326 (PMC8869309; doi:10.3390/biom12020326)
Supplement: Supplementary file 1 [file biomolecules-12-00326-s001.zip › biomolecules-1564411-SI.pdf]

biomolecules

MDPI

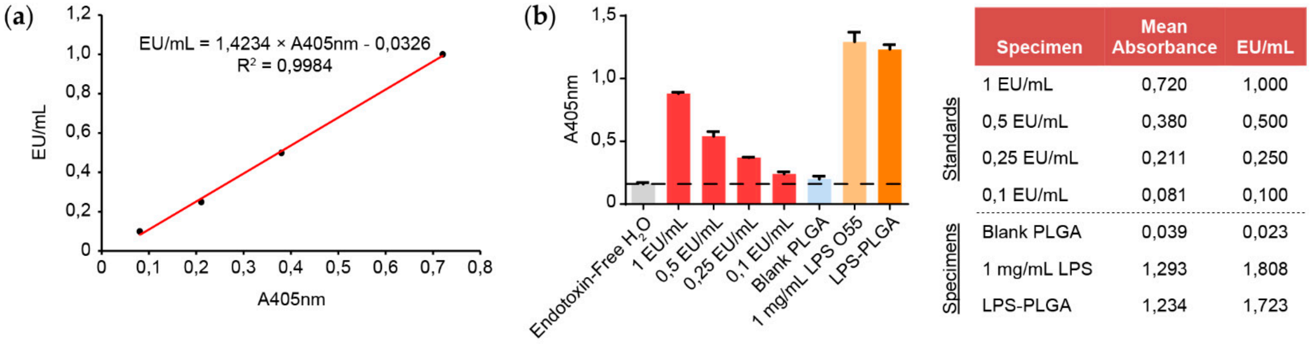

Figure S1. Endotoxin detection with the Pierce Chromogenic Endotoxin Quant Kit. (a) Using standards included in the kit, we modeled the relationship between EU/mL and absorbance at 405 nm by linear regression. (b) Using measured absorbance values from experimental reagents including 'blank'/non-LPS-encapsulated PLGA microparticles, a 1 mg/mL solution of LPS as a positive control, and LPS-encapsulated PLGA particles, we determined that the blank PLGA microparticles were endotoxin-free, and that we successfully encapsulated LPS into the PLGA microparticles (LPS-PLGA). Error bars represent SD of technical replicates.

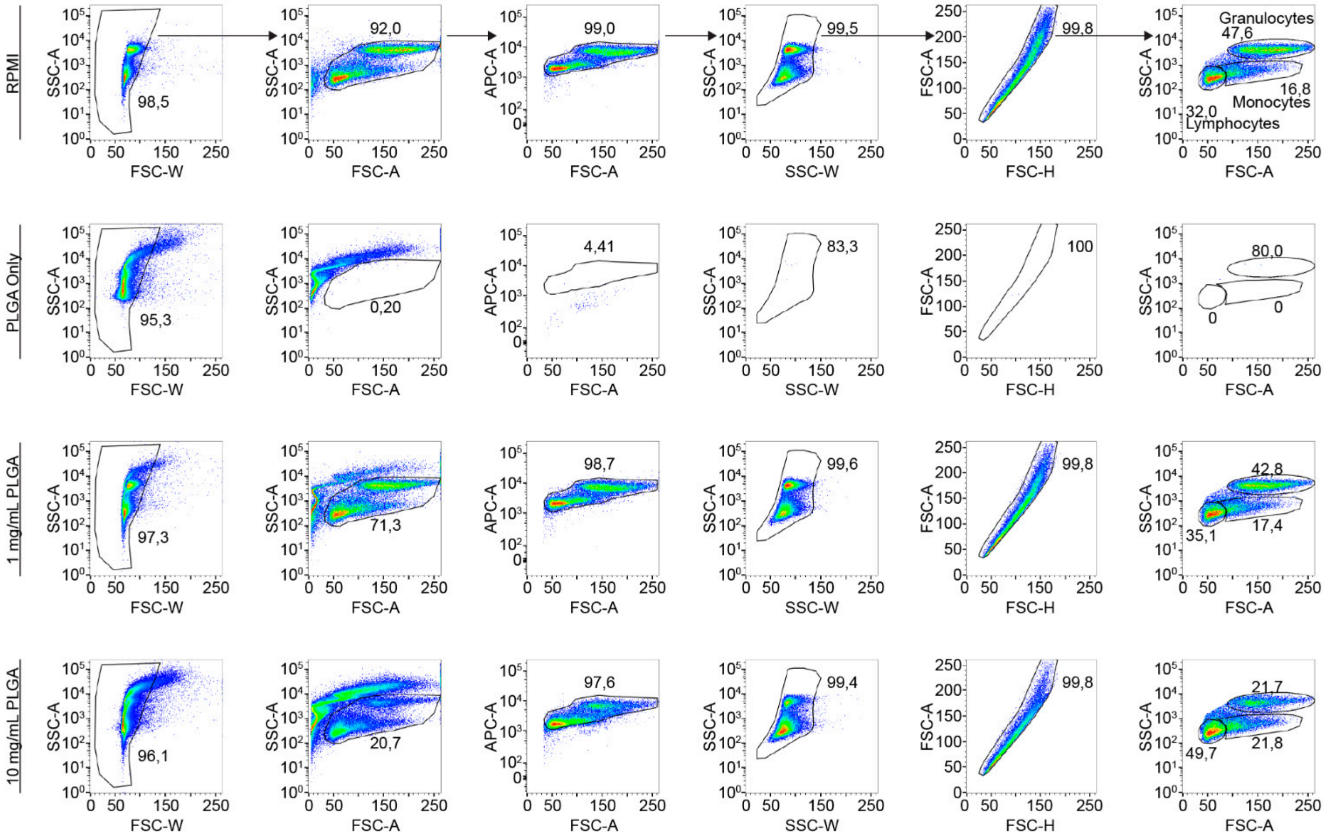

Figure S2. Gating strategies for distinguishing host cells from PLGA microparticles, and for distinguishing host leukocyte subpopulations. 25% Percoll-separated head kidney leukocytes from a healthy carp were isolated and either measured alone, or mixed with different concentrations of PLGA particles, to test if the flow cytometer can distinguish the two and exclude the polymer from downstream analyses. PLGA particles were measured alone as well to setup the gating strategy. From left to right, we applied the following gating strategy to identify host leukocytes and to differentiate peritoneal leukocyte subpopulations: 1) a plot of side scatter area (SSC-A) vs. forward scatter width (FSC-W) was applied to distinguish erythrocytes (contaminants from dissection) from

leukocytes, 2) based on the SSC-A and FSC area (FSC-A) properties of PLGA particles in the absence of host cells (second row), we created gates for host cells before an 3) APC-A vs. FSC-A plot was used to gate on NucRed Live 647⁺ live nucleated cells distinguishable from the polymer, 4) we then excluded doublets through both SSC-A vs. SSC width (SSC-W) and FSC-A vs. FSC height (FSC-H) plots, 5) finally, an SSC-A vs. FSC-A plot was set up to differentiate between lymphocytes (FSC-A^{low} SSC-A^{low}), granulocytes (FSC-A^{high} SSC-A^{high}) and monocytes (FSC-A^{high} SSC-A^{low}).

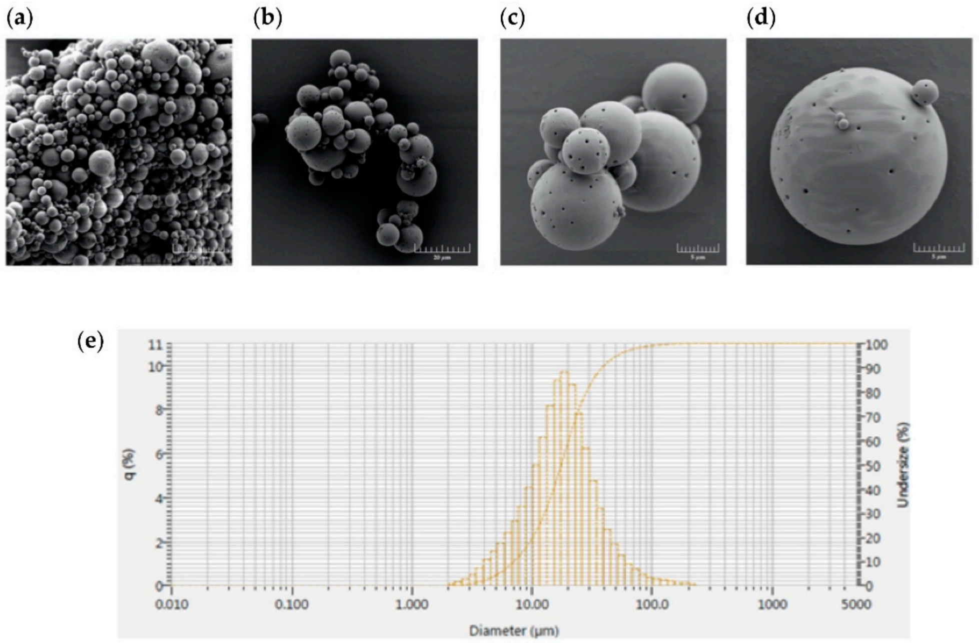

Figure S3. Scanning electron microscopy (SEM) images of the synthesized PLGA microparticles (a-d) with scale bars of 50 (a), 20 (b), or 5 μm (c-d). Size distribution of the synthesized PLGA particles (e).

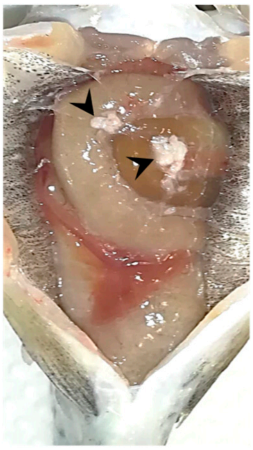

Figure S4. Fibrotic tissue in the peritoneal cavity of a fish injected 4 days prior with PLGA particles encapsulated with LPS. Although accelerated in kinetics, perhaps due to the LPS content, the formation of fibrotic tissue (indicated by black arrows) is a sign of pathology similar to that seen in the foreign body response against implanted synthetic biomaterials described in mammals. This tissue may serve to isolate and sequester clumps of PLGA microparticles that cannot be phagocytosed by infiltrating leukocytes.
